# Athletic identity transfer and integration among Saudi athlete-entrepreneurs: a qualitative exploration

**DOI:** 10.3389/fpsyg.2026.1871323

**Published:** 2026-09-15

**Authors:** Mashael Mousa Alnowaiser, Wissal Boughattas, Safiya Mukhtar Alshibani

**Affiliations:** 1 Department of Management, College of Business Administration, Princess Nourah Bint Abdulrahman University, Riyadh, Saudi Arabia; 2 Department of Physical Sports Sciences, College of Sport Sciences and Physical Activity, Princess Nourah Bint Abdulrahman University, Riyadh, Saudi Arabia

**Keywords:** athletic identity, career transition, qualitative research, Saudi Arabia, sport entrepreneurship

## Abstract

**Introduction:**

Athletic identity, the degree to which individuals define themselves through their sport role, persists long after or alongside active sporting engagement and shapes behavior, decision-making and self-concept. Despite growing interest in the relationship between sport and entrepreneurship, little is known about how this identity transfers into entrepreneurial contexts, particularly outside Western settings. This study examines how Saudi athlete-entrepreneurs experience and make sense of athletic identity transfer within the context of Saudi Arabia's Vision 2030 reforms, which have rapidly expanded the national sport sector and opened new entrepreneurial pathways for athletes.

**Methods:**

An exploratory qualitative design was adopted. Semi-structured in-depth interviews were conducted in Arabic with 24 Saudi athlete-entrepreneurs (10 men, 14 women), predominantly amateur and recreational athletes with sustained sport engagement. Data were analyzed using reflexive thematic analysis.

**Results:**

Four overarching themes of sport-derived resource transfer were identified: sport-derived personal traits, sport-derived professional skills, sport-derived athletic legacy, and sport-derived social and community values. A marked identity tension also emerged: the majority of participants reported a discrepancy between their internal athletic self-perception and their external entrepreneurial image, a pattern particularly pronounced among women.

**Discussion:**

The findings support an identity-layering rather than identity-replacement account of the sport-to-entrepreneurship transition and extend sport entrepreneurship and entrepreneurial identity theory beyond their predominantly Western boundaries. They carry implications for athlete career development programmes and sport management policy in Saudi Arabia and the broader Arab world.

## 1 Introduction

The transition from competitive sport into entrepreneurial activity has become an increasingly visible career trajectory for professional, amateur and recreational sport participants alike (Boyd et al., 2021; Hammerschmidt et al., 2022). Athletic identity, defined as the degree to which individuals define themselves through their sport role, is typically formed early in the career and reinforced through years of rigorous training, competition and affiliation with sporting communities. Its influence persists well beyond active participation, continuing to shape life choices, professional trajectories and self-concept in post-sport contexts (Boyd et al., 2021). When athletes move into entrepreneurship, this ingrained sense of self becomes consequential. It affects how they perceive opportunities, assess risk, mobilize networks and construct a legitimate entrepreneurial self.

A growing body of research demonstrates that sport-derived competencies, including resilience, discipline, teamwork, strategic thinking, leadership and goal orientation, are frequently repurposed in venture creation (Boyd et al., 2021; Hammerschmidt et al., 2022). Former elite athletes often describe their entrepreneurial journey not as an abandonment of the athletic self but as a reconfiguration of an existing identity structure toward new goals. This overlap can reduce the role conflict typically observed during career transitions, and public visibility, reputation and referent power accrued in sport can function as valuable social capital for stakeholder engagement and market entry (Boyd et al., 2021).

Despite this accumulating evidence, the empirical base remains heavily anchored in Euro-American contexts (Cardella et al., 2021; Costa and Miragaia, 2022; Massao et al., 2023). A recent integrative review identified only 12 studies offering peripheral-country perspectives out of 1,971 papers retrieved, confirming that current theory continues to reflect Eurocentric views of sport entrepreneurship (Massao et al., 2023). Non-Western settings, which differ considerably in institutional capacity, cultural expectations surrounding sport careers, gender norms and the availability of dual-career support structures, have received comparatively little attention. Existing findings are therefore rarely tested against environments in which the opportunity structure is shaped primarily by state-led transformation rather than by mature, club-based developmental pipelines (Ratten, 2021).

Saudi Arabia offers a particularly compelling setting in which to address this gap. Under Vision 2030, sport has been positioned both as an instrument of cultural enrichment and as a strategic economic sector, generating large-scale infrastructure investment, a rapidly expanding calendar of international events, new professional leagues and a historic expansion of women's participation in sport and entrepreneurship (Albujulaya et al., 2023; Aljarodi et al., 2024). These reforms have opened entrepreneurial pathways across sports technology, fitness services, events management, e-sports, merchandise and athletic training that did not exist on this scale a decade ago. Yet Saudi athletes pursue these opportunities within an ecosystem that is still maturing. Formal dual-career programmes, league-led transition curricula and institutional mentoring schemes of the kind common in Western systems (Condello et al., 2024; González-Serrano et al., 2021a; Stambulova et al., 2009) remain limited, while informal mentorship, family networks and state-supported initiatives play a proportionally larger role.

Western-centered frameworks offer limited insight into this setting, leaving a research gap that the present study seeks to fill. Three questions therefore guide the present study. How does athletic identity transfer into entrepreneurial practice when formal dual-career structures are absent? Which sport-derived traits, skills and relational resources are most actively repurposed? How do gender, family structure and cultural expectations shape this process, particularly for women whose access to competitive sport and business leadership has only recently expanded?

To address these questions, the study adopts an exploratory qualitative design, drawing on in-depth interviews with 24 Saudi athlete-entrepreneurs (10 men, 14 women). The term athlete-entrepreneur is used here in a deliberately broad sense to denote entrepreneurs with a sustained sport participation background; as detailed in the Methods section, participants were predominantly amateur and recreational athletes rather than former professional athletes, and the study therefore advances claims about elite post-sport career transition and formal dual-career pathways only in carefully qualified form. It pursues three aims. First, it extends the athlete-entrepreneur identity literature beyond its Western boundaries by offering the first qualitative account of athletic identity transfer in a Saudi and Gulf context. Second, it tests the cultural portability of established entrepreneurial identity frameworks, notably the typology of Sieger et al. (2016) and the archetype model of Sawang et al. (2025), in a setting shaped by Islamic values, familial structures and state-directed sport development. Third, it advances the taxonomy of Ratten (2023) and the conceptual framework of Hammerschmidt et al. (2022) by providing empirical evidence of sport entrepreneurship at the identity level in a non-Western context.

The paper is organized as follows. Section 2 reviews the literature on athletic identity, sport-to-business identity transitions, sport entrepreneurial ecosystems, and the Saudi Vision 2030 context. Section 3 describes the methodology. Section 4 presents the four overarching themes of sport-derived resource transfer and the gendered identity tension that cuts across them. Section 5 discusses the findings in relation to existing theory, and Section 6 draws implications for theory, policy and sport management practice in the Arab world.

## 2 Background

### 2.1 Athletic identity and its persistence beyond sport

Athletic identity is a deeply internalized dimension of self-concept that develops through sustained engagement in competitive sport. It is typically formed early in an athlete's career and consolidated over years of training, competition and socialization within sporting communities (Boyd et al., 2021; Sieger et al., 2016). Beyond a simple self-label, athletic identity carries a coherent set of values, behavioral repertoires, decision-making heuristics and psychological orientations that guide action both inside and outside the arena of competition.

A defining feature of this identity is its durability. For many athletes, the sporting self persists long after formal retirement, continuing to inform life choices, personal relationships and professional decisions (Boyd et al., 2021). When competitive engagement ends or shifts, the athletic self does not simply disappear; it is renegotiated. Social identity also interacts with individual personality traits to produce distinct founder profiles that differ in their competitive, collaborative and mission-driven orientations (Sawang et al., 2025). Understanding how this persistent identity is reconstructed during the move into entrepreneurship is therefore central to any account of athlete-led venture creation. Importantly, athletic identity is not confined to professional or elite sport. The serious leisure perspective demonstrates that sustained, committed amateur and recreational participation can generate a durable, identity-central attachment to sport, through which participants build a leisure career, acquire skills and anchor a valued social identity (Stebbins, 2007; Green and Jones, 2005). This literature provides an appropriate theoretical foundation for samples, such as the present one, whose engagement with sport is committed and identity-defining without being professional.

### 2.2 Athletic to entrepreneurial identity transitions

Analyzing the existing literature on post-sport career transitions, three analytical framings can be identified to describe how athletic identity evolves when athletes move into new occupational domains. Each points attention to a different aspect of the transition, and all three are relevant to the present study.

A first perspective, which may be termed identity displacement, treats the athletic self and the post-sport self as relatively discrete. Transition is understood as a sequential exchange in which the athletic identity is gradually set aside and replaced by a new occupational identity, such as employee, manager or entrepreneur (Costa and Miragaia, 2022). In this reading, the psychological challenge of transition lies in relinquishing the sporting self and constructing a qualitatively different one.

A second angle, which may be termed identity abrasion, emphasizes the friction produced when the athletic self and the new occupational self coexist uneasily. Retired athletes, particularly those leaving sport abruptly through injury or deselection, are frequently described as experiencing identity foreclosure, loss of status and diminished self-worth as the salience of athletic identity erodes without a stable replacement (Cota Powell et al., 2024). The emphasis is on loss, role conflict and the psychological costs of incomplete transition, a framing that has informed much of the intervention work embedded in professional leagues and collegiate systems in North America and parts of Europe (Costa and Miragaia, 2022).

A third interpretation, which may be termed identity layering, conceptualizes the post-sport self as built over rather than instead of the athletic self. Here, entrepreneurial identity is constructed as an additional, overlapping layer that draws on, rather than replaces, the competencies, dispositions and symbolic capital of the sporting career (Boyd et al., 2021; Hammerschmidt et al., 2022). Sport-derived competencies and referent power become entrepreneurial resources, and transition reconfigures rather than dismantles the athletic self.

These three approaches carry very different implications for programme design, policy and individual wellbeing. Displacement and abrasion framings tend to justify deficit-oriented interventions that treat the retired athlete as someone in need of new content, such as business skills, financial literacy or a new identity, to be supplied from outside. A layering perspective, in contrast, positions athletes as identity-rich practitioners whose existing cognitive and behavioral repertoires are already functionally applicable to entrepreneurial contexts, and whose transition support is better framed as scaffolding and redirection than as reconstruction from scratch (Boyd et al., 2021). The present study engages directly with this analytical distinction by examining which of the three framings best accounts for the experiences of Saudi athlete-entrepreneurs, where state-driven expansion of sport and entrepreneurship coincides with a weakly institutionalized dual-career infrastructure.

In examining these three framings, it is analytically important to distinguish between the different registers of “transfer” that are frequently conflated in the sport-to-entrepreneurship literature. Following identity theory (Caza et al., 2018) and the founder social identity literature (Sieger et al., 2016), the present study treats athletic identity transfer as the movement of a specific identity-level self-schema, the sense of being an athlete, into the entrepreneurial role, such that the entrepreneur continues to interpret and enact the new role through an athletic self-understanding. This is conceptually distinct from sport-derived skill transfer, which refers to the redeployment of learned competencies such as time management, strategic planning or negotiation acquired through sport (Hammerschmidt et al., 2022), from personal trait transfer, which refers to relatively stable dispositions such as conscientiousness, resilience or competitiveness that may have been reinforced by athletic experience but are not themselves identity claims from social capital transfer, which refers to the mobilization of sport-derived networks, reputation and referent power in entrepreneurial contexts (Ratten, 2015) and from entrepreneurial identity integration, which refers to how the entrepreneurial self is combined with, layered over or set against a pre-existing self-schema (Boyd et al., 2021). These registers often co-occur in practice, but the theoretical claim advanced in this paper is specifically about identity transfer and identity integration. Sport-derived traits, skills and social capital are treated as evidence of identity transfer only when participants explicitly interpret them as extensions of an athletic self-understanding rather than as generic personal qualities. The analytic implication is that not every positive quality attributed to sport constitutes evidence of athletic identity transfer and this distinction is maintained throughout the analysis and discussion.

### 2.3 Sport entrepreneurial ecosystems and the Western orientation of existing evidence

The second strand of relevant literature concerns the ecosystems within which sport entrepreneurship takes place. Studies have shown that the development of sport entrepreneurial ecosystems depends on specific combinations of human capital, infrastructure, knowledge networks and business sophistication, and that these resources are unevenly distributed across national contexts (González-Serrano et al., 2021a). Evidence also suggests that entrepreneurial orientation, particularly innovation, risk-taking and proactivity, is associated with superior performance outcomes in sport organizations, which indicates that entrepreneurial disposition is embedded not only in individuals but also in sporting cultures themselves (Escamilla-Fajardo et al., 2021). Recent work further demonstrates that sport entrepreneurs who combine strategic orientation with adaptive innovation are better positioned to sustain their ventures under market disruption (González-Serrano et al., 2023).

In Western settings, these ecosystem conditions are typically reinforced by institutional supports for dual-career development. Programmes embedded within clubs, leagues and educational institutions prepare athletes for transition through formal entrepreneurship modules, alumni mentoring schemes and seed-funding pathways linked to federations (Costa and Miragaia, 2022; González-Serrano et al., 2021a). Collegiate systems channel graduating athletes toward business endeavors upon exhaustion of eligibility (González-Serrano et al., 2021b), while professional leagues intervene systematically as players approach retirement through injury or declining performance. The scholarship grounded in these environments has been instrumental in identifying both the psychological underpinnings and the structural mechanisms of athlete-to-entrepreneur transitions.

The limitation is that this evidence base is heavily Euro-American in origin. Non-Western settings differ along several dimensions that are theoretically consequential for identity transfer. They differ in the institutional capacity of the sport system, in cultural and religious expectations attached to sport careers, in the social acceptability and structure of women's participation, in the architecture of capital allocation in emerging markets, in the role of sport business models in generating community value (Pittz et al., 2021), and in the use of sport events as mechanisms for local entrepreneurial activity and sustainable economic development (Tsekouropoulos et al., 2022). Evidence from non-Western contexts illustrates how country-specific cultural and organizational regulations produce significant disparities in flexible academic paths, financial support and individualized agreements for athletes (Condello et al., 2024). Each of these factors, in isolation and in combination, can reshape opportunity structures for former athletes in ways that Western-centric studies cannot capture. To date, these differences have been largely theorized rather than empirically examined, and the existing body of work tends to extrapolate findings from comparatively homogeneous samples (Cardella et al., 2021; Malete et al., 2022; Massao et al., 2023).

### 2.4 Sport sector transformation and entrepreneurship under Vision 2030

Saudi Arabia provides a strategic case for addressing this gap because its sport and entrepreneurship ecosystems have been reshaped within a compressed policy horizon. Vision 2030, announced in 2016, positions economic diversification, human capital development and social participation as interlocking national priorities (Albujulaya et al., 2023; Alshuwaikhat and Mohammed, 2017). Sport has been explicitly incorporated into this agenda both as a lever of cultural modernization and as a sector expected to generate employment, investment and international visibility.

The operational consequences are substantial. Large-scale investment has been directed toward stadiums, training facilities and sporting infrastructure; the country has become a recurring host of high-profile international competitions across football, motorsport, combat sports, golf and tennis; new professional leagues have been established; and commercial rights regimes around competitions have expanded rapidly. At the same time, and in a historical shift, formal participation of women in sport has been enabled and encouraged, with parallel reforms opening women's access to entrepreneurship, finance and business leadership (Aljarodi et al., 2024; Costa and Miragaia, 2022). For current and former athletes, these developments have generated entrepreneurial opportunities in domains such as sports technology, fitness enterprises, event management, e-sports, merchandising, and athletic training and consulting.

Two features of this ecosystem are particularly important for the present study. First, the Saudi sport sector remains relatively young compared with long-established Western markets and does not yet benefit from entrenched club-based entrepreneurship curricula or league-driven transition programmes. Opportunity structures are therefore emerging amid evolving institutions rather than within mature developmental pipelines. Informal mentorship, personal and family networks, and collaborations with international partners are consequently more influential than formally institutionalized dual-career programmes in facilitating athlete-led ventures. Second, cultural norms around work roles, gender and family interact dynamically with sport-based identities. Female athlete-entrepreneurs, in particular, operate in a landscape that has only recently expanded at scale and in which access to financing and representation within decision-making bodies remains uneven (Costa and Miragaia, 2022). Identity transfer from sport to entrepreneurship in this context is therefore not merely a matter of psychological adaptation; it unfolds against a backdrop of evolving policy frameworks and societal discourses that may amplify opportunities for certain groups while narrowing them for others.

### 2.5 Theoretical perspective

The study draws on identity theory, the serious leisure perspective on amateur sport participation (Stebbins, 2007; Green and Jones, 2005) and career transition literature, in which identities are viewed both as relatively stable cognitive schemas that guide behavior over time and as malleable constructs subject to reconstruction during life-course change (Boyd et al., 2021). Entrepreneurial identity research similarly frames the entrepreneur not only as someone performing particular tasks but as someone inhabiting a socially recognized role carrying meanings of autonomy, innovation and opportunity pursuit (Sieger et al., 2016). Read together, these perspectives suggest that Saudi athletes moving into entrepreneurship negotiate the boundary between a long-standing athletic identity organized around performance narratives and an emerging entrepreneurial identity shaped by market demands, cultural expectations and the macro-level incentives generated by Vision 2030.

## 3 Methodology

### 3.1 Research design

This study adopts an exploratory qualitative design grounded in identity theory and in an athletic identity persistence and layering perspective (Boyd et al., 2021; Caza et al., 2018), which conceptualizes the athletic self as an enduring identity resource that is renegotiated and layered into new roles rather than relinquished. Career transition frameworks (Stambulova et al., 2009) are drawn upon only as a point of theoretical contrast, because most participants never exited a competitive athletic career. The exploratory approach was selected due to the limited research examining how athletic identity transitions into entrepreneurial contexts, particularly in non-Western populations. Sample and self-identification characteristics are additionally summarized using simple descriptive frequencies and percentages (Tables 1, 2) to contextualize the qualitative findings; this descriptive quantification does not constitute a mixed-methods design and plays no role in the generation or interpretation of themes, which follows Braun and Clarke's (2006, 2019, 2021) reflexive thematic analysis throughout. The study is situated within an interpretivist epistemological stance, recognizing identity as socially constructed and dynamically negotiated through lived experience (Brown, 2020).

**Table 1:** Demographic profile of participants (24 Saudi athlete-entrepreneurs).

Participant	Gender	Marital status	Children	Education level	Years in sport	Years in entrepreneurship	Sport engagement	Participation in organized amateur events
P1	Female	Married	Yes	Postgraduate	Since childhood	5 years	Recreational	No
P2	Male	Married	Not disclosed	Bachelor	Since childhood	18 years	Amateur competitive	Yes
P3	Female	Married	Yes	Postgraduate	6 years	13 years	Recreational	No
P4	Female	Single	No	Postgraduate	Since age 13	1.5 years	Recreational	No
P5	Male	Married	Yes	Postgraduate	Since childhood	2.5 years	Recreational	No
P6	Female	Married	Yes	Medical Doctor (MD)	15 years	2 years & 10 months	Amateur competitive	Yes
P7	Male	Single	No	Bachelor	Since childhood	2 years	Recreational	No
P8	Male	Married	Not disclosed	Postgraduate	Since childhood	20 years	Recreational	No
P9	Female	Single	No	Bachelor	Since childhood	4 months	Recreational	No
P10	Female	Single	No	Bachelor	11 years	1.5 years	Recreational	No
P11	Female	Single	No	Bachelor	23 years	5 years	Recreational	No
P12	Female	Divorced	Yes	High school & certificates	9 years	3 years	Recreational	No
P13	Female	Single	No	Not disclosed	12 years	5 years	Recreational	No
P14	Female	Single	No	Postgraduate	11 years	7 years	Amateur competitive	Yes
P15	Female	Not disclosed	Not disclosed	Not disclosed	9 years	2 years	Amateur competitive	Yes
P16	Female	Single	No	Postgraduate	Since childhood	15 years	Amateur competitive	Yes
P17	Female	Married	Yes	Bachelor	10 years	3 years	Recreational	No
P18	Male	Married	Yes	Medical Doctor (MD)	7 years	5 years	Amateur competitive	Yes
P19	Male	Single	No	Bachelor	26 years	4 years	Recreational	No
P20	Female	Single	No	Bachelor	Since childhood	6 years	Recreational	No
P21	Male	Single	No	Bachelor	Since childhood	14 years	Amateur competitive	Yes
P22	Male	Married	Yes	Postgraduate	Since childhood	25 years	Recreational	No
P23	Male	Married	No	Postgraduate	Since childhood	11 years	Amateur competitive	Yes
P24	Male	Married	Yes	Postgraduate	30 years	25 years	Recreational	No

**Table 2:** Self-identification patterns among participants (24 Saudi athlete-entrepreneurs).

Identity category	Self-Perception (how participants see themselves)	External perception (how others see them)
Athlete	37.5% (*n* = 9)	20.8% (*n* = 5)
Entrepreneur	33.3% (*n* = 8)	50.0% (*n* = 12)
Both	29.2% (*n* = 7)	29.2% (*n* = 7)
Total	100% (*N* = 24)	100% (*N* = 24)

The study is set in Saudi Arabia, where Vision 2030 reforms have significantly expanded both the sport sector and the entrepreneurship ecosystem, creating new professional pathways for athletes (Albujulaya et al., 2023). Recent reforms related to women's participation in sport and business make this context particularly relevant for examining identity transition across gender (Aljarodi et al., 2024).

### 3.2 Participants

Purposive sampling was used to select information-rich participants. Inclusion criteria were: (a) Saudi nationals; (b) current or former athletes at professional, semi-professional, amateur or recreational level; and (c) currently engaged in entrepreneurial or leadership roles. The final sample consisted of 24 participants (10 men and 14 women). Rather than invoking data saturation as a marker of adequacy, an approach with which Braun and Clarke (2021) urge caution in reflexive thematic analysis, the sample was considered sufficient for the exploratory aims of the study because it provided information-rich accounts across gender, educational background, years of sport participation and entrepreneurial experience.

Participants were predominantly recreational and amateur athletes who engaged in sport as a consistent life practice rather than as a competitive profession. Specifically, sixteen participants engaged in sport at a recreational level, while eight (four women and four men) were amateur competitive athletes who took part in organized amateur events; none had pursued sport as a professional career (Table 1). This reflects the nature of the emerging Saudi sport ecosystem under Vision 2030, where structured professional pathways are still developing, and where sport participation among entrepreneurs is largely grounded in personal commitment rather than institutionalized athletic careers. This composition reflects purposive sampling oriented toward information power (Malterud et al., 2016) rather than a predetermined quota. Two considerations shaped it. First, individuals with sustained recreational engagement constitute the larger population of identity-invested athlete-entrepreneurs currently visible in Saudi Arabia, since organized amateur competitive structures are a more recent and still-narrower feature of the Vision 2030 sport ecosystem than recreational participation itself, the sample's skew toward recreational participants is therefore a reasonably faithful reflection of the accessible population. Second, the eight amateur competitive participants were deliberately retained, rather than excluded to produce a more homogeneous recreational-only sample, in order to test whether organized competitive experience produced a qualitatively distinct pattern of identity transfer. As reported below, the two subgroups converged rather than diverged on this measure, a result that would not have been observable without their inclusion. Accordingly, the sample is most accurately characterized as Saudi amateur and recreational athlete-entrepreneurs, and the term athlete-entrepreneur is used throughout the manuscript in this qualified sense rather than as a reference to professional or elite athletic careers. Consistent with this positioning, the findings are interpreted as evidence of identity transfer among sport participants who become entrepreneurs, not as claims about post-sport career transition, post-sport identity change or dual-career pathways among elite athletes. Studying this population offers a unique opportunity to examine how sport identity and entrepreneurial identity interact outside highly institutionalized sport systems. Table 1 presents the demographic profile of the participants.

As shown in Table 1, the sample comprised 24 Saudi athlete-entrepreneurs (14 women and 10 men), with diverse marital, parental, and educational backgrounds. Years of sporting experience ranged from a few years to lifelong engagement since childhood, and entrepreneurial experience ranged from < 1 year to 25 years, reflecting a broad spectrum of athletic and business trajectories. In terms of sport engagement, sixteen participants (66.7%) were classified as recreational athletes, none of whom had taken part in organized amateur events and eight (33.3%) as amateur competitive athletes, all of whom had. A systematic comparison of the two groups shows that they were closely aligned on the depth of their sport involvement: 43.8% of recreational participants and 50.0% of amateur competitive athletes had been engaged in sport since childhood, indicating that long-term participation characterized the sample as a whole rather than only those who competed. The groups differed more visibly in professional profile, with amateur competitive athletes reporting longer entrepreneurial experience (median 9.0 vs. 4.5 years) and more frequently holding postgraduate or medical qualifications (62.5% vs. 43.8%). Analytically, both groups contributed accounts to each of the four aggregate dimensions of identity transfer and no dimension was specific to participants who had competed in organized events, supporting the treatment of the sample as a single analytical category.

### 3.3 Data collection

Data were collected through semi-structured in-depth interviews designed to explore three domains: (a) athletic identity formation; (b) transition processes from sport to entrepreneurship and (c) identity integration within entrepreneurial leadership.

Interviews were conducted between December 2025 and February 2026 via Google Meet. Individual interviews lasted approximately 40 min on average, amounting to a cumulative total of around 16 h of recorded material across the 24 participants. The interview guide combined brief demographic items (gender and current professional role) with two open-ended core questions designed to elicit narrative accounts of identity transfer: (a) “What is the athletic legacy you have acquired from your sporting experience?” and (b) “What is the sustained impact of your sport practice on your current life and work?” Follow-up probes were used to elicit concrete examples and to explore gendered and cultural dimensions of these experiences. All interviews were conducted in Arabic to ensure participants could express identity-related concepts naturally, audio-recorded with consent, and transcribed verbatim. The semi-structured format provided consistency while allowing participants to narrate their experiences in their own terms.

### 3.4 Data analysis

Data were analyzed using Braun and Clarke's (2006, 2019, 2021) reflexive thematic analysis. This method involves reading through interview transcripts carefully, identifying meaningful patterns across participants' responses, and organizing these patterns into themes. The analysis was conducted at two levels: the semantic level, which focused on what participants directly said about their experiences, and the latent level, which looked at the deeper meanings behind their words, such as cultural expectations and identity tensions that participants may not have stated explicitly.

The analysis followed the six phases outlined by (Braun and Clarke 2006, 2019, 2021), applied as a recursive rather than linear procedure. In practice, familiarization involved repeated reading of each Arabic transcript alongside the corresponding audio recording, during which the lead analyst wrote analytic memos in Arabic capturing initial impressions, emotional tone and culturally specific expressions that would be difficult to render in English. Initial coding was conducted line by line in Arabic within a shared spreadsheet, generating short semantic codes tied to specific transcript segments together with latent-level annotations reflecting cultural expectations, gendered positioning and identity work that participants did not state explicitly. Two bilingual researchers coded the full dataset independently, while a third researcher, with expertise in entrepreneurship theory, coded a subset of transcripts to broaden interpretive perspectives. Rather than treating divergent readings as errors to be eliminated through inter-coder agreement, the team held regular interpretive meetings in which alternative codings were discussed as substantive analytic material, in keeping with Braun and Clarke's (2019) view that researcher subjectivity is a resource to be reflexively examined rather than a source of bias to be neutralized. Candidate themes were then generated by clustering conceptually related codes, reviewed against both the full dataset and the individual coded extracts, and iteratively refined through further team discussion until each theme captured a coherent and internally consistent pattern of meaning. Themes were subsequently named, defined and written up with supporting quotations and interpreted through the identity persistence and layering perspective outlined above (Boyd et al. 2021); (Caza et al. 2018). Only after themes and representative excerpts had been finalized in Arabic were the selected passages translated into English for reporting, following the translation procedure described below.

The written-up themes were subsequently organized into a thematic structure linking codes, subthemes and overarching themes, which is presented in Table 3.

**Table 3:** Data structure of identity transition from sport to entrepreneurship.

Interview excerpts	Codes	Subthemes	Overarching themes
“Patience was a big part of my journey. I started sports at 18, just following my passion... Today I own a fitness club, and I believe patience gets you to your goal.” (Participant 1)	Patience	**Patience & endurance**	**Sport-derived personal traits**
“You commit to practice, over a long time, your endurance increases... The endurance you learn from running transfers to life and business.” (Participant 16)	Endurance		
“I generally have more patience than my sister. I had patience with the workers, the contractors, calling them twenty times until they answered.” (Participant 4)	Long-term perseverance		
“Not being attached to results transferred with me to business. It made me always focus on the work I can do now... How can I be better than yesterday.” (Participant 21)	Detachment from immediate results		
“The first thing is stubbornness and determination, this transferred from sport to entrepreneurship.” (Participant 23)	Determination & positive stubbornness	**Determination & resilience**	
“In sport you fail a lot and that is something you are used to... You lose and after the loss you get up and learn from it... When I entered entrepreneurship, it was already familiar.” (Participant 23)	Accepting failure & learning from it		
“It gives you confidence because there are many things you cannot do and then you just do them... Sport is what got me to this point, nothing is too big for me.” (Participant 20)	Self-confidence	**Self-confidence & personal growth**	
“Sport is naturally within a person but it grows... There needs to be awareness about this.” (Participant 19)	Self-awareness & development		
“Discipline after practicing sport is a different experience... The marathon is a journey from point A to point B... Everything in life is the same idea.” (Participant 18)	Discipline	**Discipline & time management**	**Sport-derived professional skills**
“I learned how to manage my time because training is all about time management. You have a set time for this, a set time for that, even your nutrition.” (Participant 2)	Time management & prioritization		
“Sport definitely developed this personality... This commitment would not have grown to this level without sport... And I definitely used it in entrepreneurship.” (Participant 11)	Commitment to routine		
“Mental flexibility, dealing with setbacks, consistency, motivation these are authentic traits in both the entrepreneur and the athlete.” (Participant 3)	Mental flexibility & toughness	**Strategic thinking & mental agility**	
“He has flexibility, mental toughness, focus power, achievement power, a vision for the future, foresight for what will happen next.” (Participant 3)	Foresight & vision				
“The biggest skill I gained is balance... I balance my effort, I do not over-push or slack off... This helped me greatly in my business, balancing my words, balancing the logic of my thinking.” (Participant 9)	Balance		
“Continuous attempts... We always try in all directions... Some will succeed, some will not, and so we try to develop, improve, and learn.” (Participant 7)	Continuous trying	**Persistence & sustainability**	**Sport-derived athletic legacy to entrepreneurship**
“Discipline and calmness, if you go to the gym you will be calmer and have clarity of mind... Work after that will have more focus.” (Participant 5)	Calmness & mental clarity		
“Commitment, we can call it continuity, yes, sustainability in work.” (Participant 8)	Sustainability in work		
“Learn the simple thing and start applying it, even as a volunteer... Enter and help the person in charge with some tasks.” (Participant 22)	Willpower & patience	**Entrepreneurial drive from sport**	
“I learned the meaning of social relationships. I learned how to deal with others.” (Participant 2)	Dealing with others & building relationships	**Interpersonal skills & relationship building**	**Sport-Derived social & community impact to entrepreneurship**
“Fair competition, in business my competition is fair. It is hard to be enemies. The environment I grew up in, we compete but we do not become enemies.” (Participant 17)	Sportsmanship & fair competition	**Sportsmanship & ethical values**	
“Team sport has a lot of patience in it, it has acceptance... It is not an individual sport, so you always share the win or the loss.” (Participant 8)	Teamwork & collective winning		

Bold text indicates the second-order subthemes and overarching (aggregate) themes generated by clustering the first-order codes derived from the interview excerpts, as described in Section 4.3.

### 3.5 Trustworthiness

Several steps were taken to strengthen the interpretive quality of the findings, framed within the assumptions of reflexive thematic analysis rather than a consensus-oriented model of rigor. As noted above, divergent readings among the coders were treated as substantive analytic material rather than resolved through inter-coder agreement (Braun and Clarke, 2019). The study also provides thick description of the participants and the Saudi context so that readers can assess the transferability of the findings to other settings (Korstjens and Moser, 2018). Key decisions made during coding and theme development were documented in an analytic audit trail to keep the interpretive process traceable (Nowell et al., 2017). The lead researcher maintained a reflexive journal throughout data collection and analysis to record shifting assumptions, emotional responses to the data and the influence of her own athletic and cultural background on the interpretation, and direct quotations from participants are used in reporting the findings to ground the interpretive claims in the data (Olmos-Vega et al., 2023).

### 3.6 Researcher positionality

The research team consisted of three researchers, one of whom is an athlete. This provided cultural sensitivity and familiarity with athletic identity in the Saudi context. To manage potential bias, the lead researcher maintained a reflexive journal documenting assumptions and interpretive decisions throughout data collection and analysis. The diversity of expertise within the team spanning athletic experience, entrepreneurship research, and qualitative methodology and quantitative methodology provided multiple analytical perspectives. One team member's additional background in descriptive quantitative summarization informed the frequency and percentage reporting of sample and self-identification characteristics in Tables 1, 2 and in Section 3.2, but no quantitative methodology was applied to the thematic analysis itself, which remained entirely qualitative and interpretive throughout.

### 3.7 Translation

Because interviews were conducted and coded in Arabic, only the passages selected for inclusion in the manuscript were translated into English, so that cultural and linguistic nuance was preserved throughout the analytical process. A forward-translation and back-translation procedure was applied to all quotations included in the manuscript: an initial English translation was produced by one bilingual researcher, back-translated into Arabic by a second bilingual researcher who had not seen the original transcript, and any discrepancies between the original and back-translated versions were discussed by the full team until conceptual equivalence was reached. Translation prioritized conceptual equivalence over literal rendering, and explanatory notes were provided where Arabic expressions lacked direct English equivalents.

### 3.8 Ethical considerations

Ethical approval was obtained from Princess Nourah Bint Abdulrahman University (IRB Log Number: 26-0026). All participants provided written informed consent and were informed of their right to withdraw at any stage. Data were stored on password protected devices accessible only to the research team.

## 4 Findings

### 4.1 Self-identification of participants

The findings are reported in two tiers. The first tier presents identity-level findings: participants' self-identification (Section 4.1), the gendered identity tension (Section 4.2) and the strategies through which the athletic identity is integrated into entrepreneurial practice (Section 4.4). The second tier presents the sport-derived resources, comprising traits, skills, legacy and values, that this persistent athletic identity transmits into entrepreneurial work (Section 4.3). Consistent with the distinction drawn in Section 2.2, these resources are treated as evidence of what the athletic identity carries, not as identity transfer in themselves. A notable pattern emerged when participants were asked how they identify themselves compared to how others perceive them. Of the 24 participants, 37.5% self-identified primarily as athletes, 33.3% as entrepreneurs, and 29.2% as both. In contrast, when asked how others perceive them, 50% reported being seen predominantly as entrepreneurs, 29.2% as both, and only 20.8% as athletes. Significantly, 62.5% of participants reported a discrepancy between their self-perception and how they are perceived by others. The most prevalent pattern within this gap involved participants who are viewed by society as entrepreneurs yet continue to identify themselves primarily as athletes. Only 37.5% reported alignment between their self-identity and external perception, as summarized in Table 2.

This finding is consistent with the literature suggesting that a strong athletic identity remains deeply embedded even after individuals transition into new careers (Caza et al., 2018), and that the outward professional image does not necessarily reflect the internal self-concept. This identity tension provides an important foundation for understanding the four overarching themes that follow, which illustrate how this enduring athletic identity actively manifests in the entrepreneurial practices, traits, and values of the participants.

### 4.2 Gender differences in athletic identity and transferred traits

A closer examination of the data revealed notable gender-based patterns in how female participants experienced the transfer of athletic identity into entrepreneurship. Among the 14 female participants, the majority self-identified primarily as athletes, yet most were perceived by others as entrepreneurs. This internal attachment to the athletic self was articulated most clearly by Participant 11, who reflected: “*I have always seen myself as an athlete, even when I stop practicing for a while… I may define myself as an athlete more than as an entrepreneur. I expected people to see me only as an entrepreneur, but, thank God, they see me as both.”* Her account captures a directional gap that characterized the female sample: an externally ratified entrepreneurial role that had not displaced an internally anchored athletic self. This pattern was more pronounced among women than men, with the majority of female participants reporting such a mismatch compared to half of the male participants (*n* = 10) suggesting that female Saudi athlete-entrepreneurs experience a greater tension between their internal athletic identity and the external entrepreneurial image imposed by society (Knoppers and McDonald, 2010; Ratten, 2018).

For several female participants, this tension was explicitly linked to the social reality of being a woman in contexts where their athletic presence was contested. Participant 15, who practiced a male-dominated combat sport, described her trajectory as a dual struggle shaped by her gender: “*As a woman entering a space like the cage, the struggle was not only within myself to practice this sport - there were people actively fighting against it, not wanting a woman to emerge and stand out in this field. It took me a long time to reach the stage where I can now stand and speak without being affected by anyone.”* Her narrative makes visible a layer of identity work that male participants did not describe: the athletic identity had to be defended and earned against a gendered social order before it could be sustained. This aligns with research documenting the invisibility and delegitimization of women's athletic identities in male-coded environments (Knoppers and McDonald, 2010).

Gendered experience also emerged at the embodied level. Participant 12 drew a direct connection between her female physiology and her need for the structuring discipline that sport provided: “*We as women have hormones, factors, pregnancy, childbirth… even if I am naturally disciplined, I could not, I could not continue. I acquired commitment and discipline only from the moment I entered the world of Pilates.”* Here, sport is not simply a source of transferable skill, it becomes a stabilizing structure that compensates for the cyclical and life-course realities of being a woman and entrepreneurship inherits that stability. This embodied reading of identity transfer extends existing theoretical accounts, which have largely treated gendered identity resources at the cognitive or social level, rather than as an embodied negotiation of the female body across athletic and entrepreneurial domains.

Beyond self-identification, gender differences also emerged in the specific traits and values participants described as being transferred from sport to entrepreneurship. Female participants more frequently emphasized internal and self-regulatory qualities such as patience, endurance, discipline, commitment and mental flexibility. Participant 1's reflection exemplifies this orientation: “*Patience was a big part of my journey… sport taught me patience, endurance, and persistence… and I believe patience takes you to your goal.”* The language here is inward-facing, grounded in self-cultivation rather than in external competition. Male participants, by contrast, placed greater emphasis on outward-facing and competitive attributes, including determination, perseverance, sportsmanship, fair competition and relationship building. Notably, both groups identified discipline and commitment as central to their entrepreneurial practice, suggesting a shared foundation regardless of gender. However, the divergence in other areas points to gendered pathways of identity transfer, where women draw more heavily on the psychological and cognitive dimensions of their athletic experience, while men leverage the social and competitive dimensions (Ronkainen et al., 2016; Brewer and Cornelius, 2001).

The psychological function of sport was particularly visible in how female participants described its role as an emotional and cognitive regulator within the pressures of their entrepreneurial and professional lives. Participant 16 captured this function directly: “*With final exams and other external pressures, sport is truly the outlet… if I go to the club, I become calmer, with clarity of mind, and even the work afterwards is more focused.”* Here, sport is not merely a transferable skill set but an ongoing regulatory resource that sustains the cognitive and emotional capacities female participants drew upon in their entrepreneurial roles. Taken together, these narratives suggest that gender in this study is not a demographic variable but a substantive identity resource and constraint: being a woman shaped the resistance female participants encountered, the embodied realities they navigated, and the psychological functions sport performed for them (Costa and Miragaia, 2022).

### 4.3 Athletic identity persistence and transfer

The thematic analysis of participants narratives was guided by an identity persistence and layering perspective (Boyd et al., 2021; Caza et al., 2018), which conceptualizes the relationship between athletic and entrepreneurial roles as a structured process in which pre-existing identity resources are renegotiated rather than abandoned. Working within this theoretical lens, the analysis followed Braun and Clarke's (2006, 2019, 2021) six-phase reflexive procedure and moved iteratively across three levels of abstraction.

At the first level, meaningful segments of participants accounts were coded inductively, staying close to the data and preserving participants own language. This produced 21 codes capturing specific traits, behaviors and orientations that participants explicitly linked to their sporting experience - for example “patience,” “endurance,” “detachment from immediate results,” “accepting failure,” “balance” and “fair competition.” These codes represent the raw identity resources that athletes described carrying from sport into entrepreneurship.

At the second level, the 21 codes were compared with one another through an iterative process of constant comparison, in which codes sharing similar conceptual meaning were clustered together, while those differing in orientation were separated. Through this process, conceptually related codes were grouped into 9 subthemes that reflected coherent psychological and behavioral patterns across participants. For instance, codes such as “patience,” “endurance” and “long-term perseverance” were consolidated under the theme Patience and Endurance; “discipline,” “time management” and “commitment to routine” were grouped under Discipline and Time Management and “mental flexibility,” “foresight” and “balance” were combined into Strategic Thinking and Mental Agility. The move from 21 codes to 9 themes allowed the analysis to capture shared patterns across participants while maintaining fidelity to the specificity of their individual transition experiences.

At the third level, the nine subthemes were further synthesized by examining how they related to one another at a broader conceptual level. Themes that reflected inward, self-directed qualities (such as Patience and Endurance, Determination and Resilience and Self-Confidence and Personal Growth) were interpreted as belonging to the same underlying domain, the transfer of personal psychological attributes. Similarly, themes reflecting structured work-related capacities (Discipline and Time Management, Strategic Thinking and Mental Agility) pointed to a distinct domain of professional skill transfer. Through this interpretive synthesis, the nine subthemes were consolidated into the four overarching themes presented in Table 3. Several coding decisions warrant explicit justification because their placement is not self-evident from the excerpt alone. For example, the code “detachment from immediate results” (Participant 21) was placed under Patience and Endurance because participants consistently framed non-attachment to short-term outcomes as an extension of the same long-horizon orientation described in codes such as “long-term perseverance” and “endurance” that is, as the temporal counterpart of endurance rather than as a discrete self-regulatory skill. A process-orientation or non-attachment reading would have been equally defensible and the alternative was actively discussed by the team, the Patience and Endurance placement was ultimately retained because participants themselves consistently linked it back to the endurance developed through sustained athletic practice rather than to mindfulness or emotion-regulation vocabularies. Similarly, “calmness and mental clarity” (Participant 5) could have been placed under an emotional-regulation or self-management theme, it was located under Sport-Derived Athletic Legacy to Entrepreneurship because Participant 5 and others in this cluster explicitly attributed this calmness to the ongoing practice of sport (“if you go to the gym you will be calmer”) framing it as an athletic legacy that continued to be produced by sport rather than as an internalized personal trait that was carried away from it. The team documented these interpretive choices in an audit trail so that alternative readings remain visible to readers.

### 4.4 Strategies for integrating athletic identity into entrepreneurship

The findings revealed four key strategies through which participants actively transferred their athletic resources into their entrepreneurial practice. All four strategies operate in a single, consistent direction from sport to entrepreneurship rather than through mutual or bidirectional exchange. Representative quotes, participants and the direction of each strategy are summarized in Table 4.

**Table 4:** Integration strategies: participant quotes and transfer direction.

Strategy	Representative quotes	Participants	Direction of transfer
1. Maintaining sport as a daily routine	“*It is impossible for a day to pass without training… I never start anything in my day before any calls, before any correspondence, before any emails I start with training. This routine is as essential to me as my prayer.”* (P1)	P1, P2, P5, P16, P17	Sport → Entrepreneurship
	“*I start my day with energy… If I don't go to the club, people tell me, ‘You look different today, you didn't go, did you?' Sport is truly my outlet.”* (P16)		
	“*I am always keen to ask them: how are you, we haven't seen you last week, how is your knee, how is your shoulder… These things became part of me I used to do them when I was a trainer, now I still do them as an owner.”* (P17)		
2. Leveraging athletic experience to build the business	“*Over time, I trained more and developed myself and obtained certified credentials until I became a sports trainer. Then I thought, why not turn this passion into something bigger? So I started my own project. Today, I am the owner of Hala Fitness Club.”* (P1)	P1, P12, P13, P17	Sport → Entrepreneurship
	“*I built good friendships, I met people in the sport field I could not have met through any other means… I built my team, Wayran, and I am proud of it.”* (P17)		
	“*Running a Pilates studio taught me to take responsibility for outcomes responsibility for every decision, for everything from A to Z.”* (P12)		
3. Conscious reflection and self-awareness	“*Honestly, I have not studied commerce, business, or management but I have learnt everything from sport, and these lessons have been reflected in me later.”* (P19)	P19, P20, P21	Sport → Entrepreneurship
	“*Detachment from results transferred with me into business it made me always focus on the work I can do now, and on how I can be better than I was yesterday, even by one in a million.”* (P21)		
	“*These things give you confidence without you even noticing… my goal was to build something of my own, something from myself.”* (P20)		
4. Using sport experience to navigate business challenges	“*In business, it is the same story you find this path blocked, and you can take another path that gives you the same result, and you discover it even gives you a better result than the first. This is exactly what sport taught me.”* (P9)	P2, P9, P16	Sport → Entrepreneurship
	“*The endurance you learn from running transfers to life and business… if you can bear the pressure of sport, it helps you bear other pressures too.”* (P16)		
	“*In business, some projects succeed when you are patient, when you persist, when you find alternatives rather than stopping at the nearest obstacle. This was already present in sport.”* (P2)		

#### 4.4.1 The first strategy was maintaining sport as a daily routine

Several participants described sport not as a peripheral activity but as a structural anchor of their daily entrepreneurial life, consistently practicing it before engaging with any business task. This pattern illustrates how sport became a non-negotiable daily practice that fuels entrepreneurial performance rather than competing with it (Stebbins, 2007).

#### 4.4.2 The second strategy involved leveraging athletic experience to build the business directly

Several participants converted their athletic background into entrepreneurial ventures for example, founding fitness clubs, sports projects for girls, or sports product brands. In such cases, the business became a direct institutional extension of the athletic identity, echoing Boyd et al.'s (2021) account of athletes exploiting their accumulated sport-derived resources through entrepreneurial ventures.

#### 4.4.3 The third strategy was conscious reflection and self-awareness

Several participants described sport as the primary school through which they developed their entrepreneurial mindset, recognizing this transfer consciously and intentionally. This illustrates identity work in action: participants were not passively carrying traits forward, but actively interpreting sport as a legitimate source of entrepreneurial knowledge (Caza et al., 2018).

#### 4.4.4 The fourth strategy was using sport experience to navigate business challenges

Participants frequently drew on embodied sport experiences to make sense of and overcome entrepreneurial obstacles comparing business setbacks to athletic training, or drawing parallels between physical endurance and business resilience. This pattern aligns with Ratten's (2015) argument that athletes develop cognitive frameworks through sport that they subsequently redeploy in entrepreneurial decision-making.

## 5 Discussion

This study aimed to examine how Saudi athlete-entrepreneurs experience, make sense of, and strategically deploy their athletic identity within entrepreneurial contexts, a question whose significance is amplified by the rapid structural transformation underway in Saudi Arabia through Vision 2030. Drawing on in-depth qualitative interviews with 24 Saudi athlete-entrepreneurs, predominantly amateur and recreational athletes, the findings converge around four overarching themes of sport-derived resource transfer and a pervasive tension between internal self-perception and external social recognition. Taken together, these results both confirm and extend prior theoretical work on athlete entrepreneurship, while raising substantive questions about the cultural portability of frameworks that have been developed almost exclusively in Western contexts (Cardella et al., 2021; Malete et al., 2022).

The most important finding of this study is the extent to which athletic identity endures as the primary self-referential frame for individuals who are, by any external measure, functioning entrepreneurs. Athletic identity remained salient for the majority of participants (*n* = 16; 66.7%) who identified either primarily as athletes (*n* = 9; 37.5%) or as both athletes and entrepreneurs (*n* = 7; 29.2%) even after sustained engagement in business. At the same time, 62.5% reported a discrepancy between their internal self-concept and how others perceive them. This persistence of athletic identity is consistent with the foundational argument in the identity transition literature that strong pre-existing identities do not dissolve upon role change but persist, often beneath conscious awareness, shaping cognition and behavior long after formal exit from sport (Boyd et al., 2021). What the present study adds, however, is evidence that this persistence is not simply a transitional artifact; it appears to be a structurally stable feature of how athlete-entrepreneurs in this context organize their sense of self.

The directional character of the gap is theoretically important. Participants were not struggling to be recognized as entrepreneurs while privately mourning an athletic past; rather, they were accurately perceived by their social environment as entrepreneurs while continuing to anchor their self-concept in sport. This asymmetry suggests something more precise than identity lag. It points toward a layered identity structure in which the entrepreneurial role has been successfully performed and socially ratified without displacing the foundational athletic self-schema. This is closer conceptually to identity layering than identity replacement, a distinction that sequential substitution models do not capture well. (Boyd et al. 2021) described this process as the formation of an overlapping identity rather than a wholly new one, and the present findings offer direct empirical support for that formulation in a non-Western context.

The gendered dimension of this tension warrants particular attention. Female participants reported the identity gap with greater frequency and intensity than male participants, a pattern that is consistent with (Costa and Miragaia 2022) study, who documented persistent cultural barriers facing women in sport entrepreneurship, including the deep-seated association of sport with masculinity and the limited representation of women in entrepreneurial decision-making structures. For women in Saudi Arabia, where formal participation in sport has expanded only recently under Vision 2030 reforms, the persistence of an athletic self-concept may serve a protective and affirming function. Continuing to identify as an athlete in a social environment that has not historically endorsed that identity for women may represent an act of psychological self-preservation as much as a motivational orientation. This interpretation is consistent with the broader argument by (Aljarodi et al. 2024) that women entrepreneurs in Saudi Arabia navigate a shifting but still socially mediated landscape in which identity resources take on distinctive defensive functions not visible in more established contexts. This directional asymmetry of the gap also invites a structural reading. Under Vision 2030, the entrepreneurial role has acquired considerable social prestige as a publicly endorsed contribution to national economic diversification, whereas an enduring athletic identity, particularly for women, has been shaped through years of private, often contested effort. The observation that participants are externally recognized as entrepreneurs while continuing to anchor their self-concept in sport may therefore reflect not a mismatch to be corrected but a functional division of identity labor: entrepreneurship supplies external legitimacy, while athletic identity remains the private, hard-won source of resilience on which that legitimacy is built.

On the other hand, the four overarching themes identified through the thematic analysis, comprising sport-derived personal traits, sport-derived professional skills, sport-derived athletic legacy, and sport-derived social and community values, collectively advance the understanding of what athletes carry from sport into entrepreneurship and how that transfer operates. Consistent with the definitional distinction drawn in Section 2.2, these four overarching themes are interpreted as sport-derived resources transmitted by a persistent athletic identity, rather than as identity transfer *per se*; the identity-level evidence of the study lies in the self-identification patterns and the internal-external gap reported in Section 4.1. While prior work has generally acknowledged that athletes bring resilience, discipline, and competitiveness to entrepreneurial settings (Boyd et al., 2021; Escamilla-Fajardo et al., 2021), the present findings disaggregate these broad characterizations into a more differentiated structure and, critically, shed light on the mechanisms through which each dimension is recontextualized.

The dimension of sport-derived personal traits, encompassing patience, endurance, determination, self-confidence, and the capacity to learn from failure, aligns with the entrepreneurial resilience arguments advanced by (González-Serrano et al. 2023), who found that resilience, in combination with innovation and strategic orientation, was the most consistent predictor of sport entrepreneur performance under conditions of disruption. What the current study adds is attention to mechanism. Participants did not describe these traits as remembered and then consciously applied; they described them as physiologically and cognitively encoded through years of repetitive athletic practice. This suggests a form of embodied knowledge transfer that cognitive or motivational frameworks alone do not adequately theorize, and that extends the understanding of how athletic experience shapes entrepreneurial cognition in ways that go beyond explicit skill transfer.

The dimension of sport-derived professional skills, anchored in discipline, time management, strategic thinking, and mental agility, speaks most directly to the competency arguments advanced in the sport entrepreneurship literature. Hammerschmidt et al. (2022) proposed that sport entrepreneurship involves a distinctive process of opportunity identification and value creation rooted in the unique characteristics of sporting experience, and the present findings ground that proposition in specific narrative evidence. Participants' accounts suggest that what is being transferred is not simply a portfolio of skills but an organizing cognitive schema through which entrepreneurial challenges are interpreted and navigated. This distinction matters for programme design: if the transfer is primarily schematic rather than technical, interventions that focus on credential mapping or business knowledge acquisition may miss the more fundamental cognitive reorientation that is actually occurring.

The dimension of sport-derived athletic legacy is the most culturally specific of the four. Participants who converted their athletic background directly into business ventures, founding fitness clubs, sports product brands, or girls' sports programmes, were not simply exploiting a market opportunity. They were institutionalizing their athletic identity, making the business the medium through which the athletic self could continue to exist and generate value after or alongside active sporting engagement. This pattern echoes what (Boyd et al. 2021) described as athletes leveraging accumulated sporting resources through entrepreneurial ventures, but it also connects to the broader argument of (Ratten 2021) that sport creates distinctive ecosystem conditions through knowledge spillovers rooted in cultural identity and emotional attachment. In the Saudi context, where sport entrepreneurship is simultaneously an individual career strategy and an act of alignment with Vision 2030 objectives, this fusion of personal legacy and national purpose takes on additional significance.

Finally, the dimension of sport-derived social and community values, encompassing sportsmanship, ethical competition, teamwork, and relationship-building, is perhaps the least theorized aspect of athletic-to-entrepreneurial identity transfer in the existing literature. The participants' emphasis on fair competition as a business principle and collective winning as an organizational model suggests that the ethical and relational grammar of sport carries substantial weight in how these entrepreneurs understand their responsibilities to stakeholders and communities. This corroborates with (Pittz et al. 2021) study, who affirms that sport-based business models generate significant community value that conventional economic metrics fail to capture, and with (Tsekouropoulos et al. 2022), who demonstrated that sport events stimulate local entrepreneurial engagement through normative as well as economic mechanisms. The present findings extend these insights to the identity level by showing that community-oriented values are not merely structural features of sport organizations but are deeply internalized by individual practitioners and carried forward into their entrepreneurial conduct.

From another perspective, a study of this kind cannot be fully interpreted without sustained attention to the structural context in which participants' identity narratives were formed and enacted. Vision 2030 is not merely a policy backdrop; it is an active identity-shaping force. The expansion of the sport sector, the opening of professional pathways for women, and the state-level endorsement of entrepreneurship as a national priority have together produced a context in which athletic and entrepreneurial identities are ideologically reinforced as complementary rather than sequential or competing. This stands in stark contrast to the Euro-American settings that dominate the existing literature, where sport careers are typically framed as inherently temporary and where the move into entrepreneurship follows well-trodden institutional pathways (González-Serrano et al., 2021a; Costa and Miragaia, 2022).

At the same time, the Vision 2030 context is characterized by the relative immaturity of the institutional infrastructure supporting the athletic-to-entrepreneurial transition. Unlike the structured dual-career systems embedded in Western collegiate or professional sport organizations (Boyd et al., 2021), Saudi athlete-entrepreneurs largely navigated their transitions through informal networks and personal initiative. The absence of formalized mentorship pipelines, sport federation-linked entrepreneurship curricula, or dedicated funding instruments for athlete-led ventures means that identity transfer in this context is largely self-managed. The four strategies participants described, namely maintaining sport as daily routine, building businesses from athletic expertise, practicing conscious self-reflection, and drawing on embodied sport experience to interpret business challenges, are each compensatory mechanisms for structural support the ecosystem does not yet provide. This finding advances the argument of (González-Serrano et al. 2021a) on sport entrepreneurial ecosystems by demonstrating that under conditions of rapid policy-driven change, individual agency and identity resources can function as partial substitutes for institutional infrastructure, at least in the early stages of ecosystem formation.

The gender dimension of the Saudi context deserves additional emphasis. (Malete et al. 2022) demonstrated that context matters substantially for how sport-based entrepreneurial capacities develop, with country-level differences producing meaningful variation in outcomes. The present findings extend this insight by showing that gendered cultural dynamics within a single national context can produce divergent identity transfer patterns even within a homogeneous sample. Female participants' greater reliance on internal, self-regulatory traits and their more pronounced identity gap both reflect the specific conditions of women's sport entrepreneurship in Saudi Arabia, where the social ratification of the entrepreneurial role has outpaced the cultural legitimation of the underlying athletic identity. Sawang et al. (2025), whose construct the model maps of social identity against personality traits, offers a partial theoretical framework for this dynamic, though the present findings suggest that culturally specific gender norms introduce a layer of variation that archetype-based models require further development to accommodate.

Concerning theoretical Contributions and Implications for Practice, this study makes several interconnected contributions to the theoretical literature on athletic identity, entrepreneurial identity, and sport entrepreneurship. Most directly, it advances the conceptual vocabulary available for describing the identity dynamics of athlete-entrepreneurs. The identity layering model implied by the findings, in which entrepreneurial identity is constructed over rather than in replacement of athletic identity, offers a more empirically adequate account of career transition than the sequential substitution frameworks that have dominated the field. It also aligns with the broader identity theory perspectives that Sieger et al. (2016) drew on in developing their founder social identity framework, which demonstrates that multiple identity orientations coexist and jointly shape entrepreneurial behavior, rather than resolving into a single dominant identity over time.

The study also contributes to the growing body of work questioning the universality of entrepreneurial identity frameworks developed in Western traditions. The emphasis participants placed on ethical competition, collective contribution, and the alignment of personal ambition with community benefit reflects an identity grammar that is not fully captured by frameworks centered on individual agency and market-facing opportunity recognition. Ratten (2023) has called for greater recognition of the diversity of entrepreneurship sub-types and the inadequacy of universal definitions, and the present findings respond to that call by offering empirically grounded evidence of how cultural context, in this case the intersection of Islamic values, familial structures, and state-directed transformation, shapes the content of entrepreneurial identity in ways that existing typologies do not fully address.

Beyond these general contributions, the findings suggest several context-specific ways in which identity transfer is shaped by the Saudi setting and it is these that carry the strongest theoretical weight. First, identity transfer here is embedded in a state-led sport development trajectory. Vision 2030 has simultaneously constructed the sport sector as a strategic economic domain and legitimated entrepreneurship as a national contribution, so that participants' movement from sport into venture creation is not experienced as a private career pivot but as alignment with a publicly endorsed developmental project. Existing sport entrepreneurship theory, which is grounded in mature club-based and collegiate pipelines, does not model this state-mediated scaffolding of identity transfer. Second, the data indicate that for female participants in particular, entrepreneurial identity is externally legitimized in ways that athletic identity is not, especially given the historically contested status of women's competitive sport in the country. Several accounts suggest that sport functions as a private, hard-won source of identity continuity while entrepreneurship functions as a publicly ratified role, a division of identity labor that is not visible in Western studies where both roles enjoy comparable social endorsement. Third, the absence of formal dual-career structures reshapes the transfer process itself. Where Western athletes typically encounter institutionalized transition programmes, mentoring schemes and federation-linked funding at the point of exit from sport, Saudi participants describe transfer as largely self-managed through routine, embodied practice and personal reflection, so that identity resources partially substitute for the institutional infrastructure the ecosystem does not yet provide. Taken together, these three points sharpen the theoretical claim of the paper: what is genuinely novel in the present findings is not that sport-derived qualities are useful in entrepreneurship, but that identity transfer under state-led ecosystem formation, gendered legitimacy asymmetries and weak dual-career institutionalization follows a pattern that current Western-grounded theory does not adequately capture.

For practitioners and policymakers, the implications are equally substantive. Career transition programmes designed for Saudi athlete-entrepreneurs should begin from the recognition that these individuals are not blank-slate novices entering entrepreneurship for the first time; they are identity-rich practitioners who have already developed sophisticated cognitive and behavioral repertoires that are functionally applicable to entrepreneurial contexts. Programme design that acknowledges and scaffolds this existing capital, rather than treating athletes as deficient in business knowledge that must be supplied from scratch, is likely to be both more effective and more respectful of participants' self-understanding. The finding that female participants face stronger disparities between self-perception and social recognition has direct implications for gender-responsive career support within the Saudi sport sector, a point that both (Costa and Miragaia 2022) have argued on structural grounds.

## 6 Conclusion

This study demonstrates that athletic identity transfer among Saudi athlete-entrepreneurs is not a simple competency exchange, but a deeply layered process shaped by personal history, cultural context, and structural opportunity. The four dimensions identified, alongside the pervasive self-perception gap, both confirm existing theoretical propositions and advance them by introducing identity layering as an alternative to sequential substitution models, documenting culturally specific transfer dynamics under Vision 2030, and revealing gendered incongruences between internal self-concept and external recognition. Together, these findings point toward a more inclusive theoretical framework for understanding athletic identity in entrepreneurial life beyond Western sport contexts.

## 7 Directions for future research

Three priorities emerge from this study. First, comparative research is needed to test whether the four dimensions of identity transfer identified here hold across other non-Western contexts, particularly in African and East Asian settings where sport entrepreneurship is growing but remains under-researched. Second, a study designed explicitly around gender as an organizing variable, and based on a larger sample of female athlete-entrepreneurs, is needed to explore the mechanisms through which gender mediates identity transfer in contexts of rapid social change, building on the gendered dynamics this study has identified but could not fully investigate given the limits of the current sample. Third, because the sample consisted predominantly of amateur and recreational athlete-entrepreneurs, research involving professional and elite athletes is required before these findings can be extended to formal post-sport career transition and dual-career contexts.

## Data Availability

The original contributions presented in the study are included in the article/supplementary material, further inquiries can be directed to the corresponding author.
